# Hypothesis on Skeletal Muscle Aging: Mitochondrial Adenine Nucleotide Translocator Decreases Reactive Oxygen Species Production While Preserving Coupling Efficiency

**DOI:** 10.3389/fphys.2015.00369

**Published:** 2015-12-16

**Authors:** Philippe Diolez, Isabelle Bourdel-Marchasson, Guillaume Calmettes, Philippe Pasdois, Dominique Detaille, Richard Rouland, Gilles Gouspillou

**Affiliations:** ^1^INSERM U1045 - Centre de Recherche Cardio-Thoracique de Bordeaux and LIRYC, Institut de Rythmologie et Modélisation Cardiaque, Université de Bordeaux, CHU de BordeauxPessac, France; ^2^CHU de Bordeaux, Pôle de Gérontologie CliniqueBordeaux, France; ^3^Résonance Magnétique des Systèmes Biologiques, UMR 5536 Centre National de la Recherche Scientifique, Université de BordeauxBordeaux, France; ^4^Division of Cardiology, Department of Medicine, David Geffen School of Medicine, University of California, Los AngelesLos Angeles, CA, USA; ^5^Département des Sciences de l'activité Physique, Université du Québec À MontréalMontréal, QC, Canada

**Keywords:** adenosine nucleotide translocator, mitochondrial membrane potential, muscle energetics, oxygen free radicals, mitochondria, skeletal muscle aging

## Abstract

Mitochondrial membrane potential is the major regulator of mitochondrial functions, including coupling efficiency and production of reactive oxygen species (ROS). Both functions are crucial for cell bioenergetics. We previously presented evidences for a specific modulation of adenine nucleotide translocase (ANT) appearing during aging that results in a decrease in membrane potential - and therefore ROS production—but surprisingly increases coupling efficiency under conditions of low ATP turnover. Careful study of the bioenergetic parameters (oxidation and phosphorylation rates, membrane potential) of isolated mitochondria from skeletal muscles (gastrocnemius) of aged and young rats revealed a remodeling at the level of the phosphorylation system, in the absence of alteration of the inner mitochondrial membrane (uncoupling) or respiratory chain complexes regulation. We further observed a decrease in mitochondrial affinity for ADP in aged isolated mitochondria, and higher sensitivity of ANT to its specific inhibitor atractyloside. This age-induced modification of ANT results in an increase in the ADP concentration required to sustain the same ATP turnover as compared to young muscle, and therefore in a lower membrane potential under phosphorylating—*in vivo*—conditions. Thus, for equivalent ATP turnover (cellular ATP demand), coupling efficiency is even higher in aged muscle mitochondria, due to the down-regulation of inner membrane proton leak caused by the decrease in membrane potential. In the framework of the radical theory of aging, these modifications in ANT function may be the result of oxidative damage caused by intra mitochondrial ROS and may appear like a virtuous circle where ROS induce a mechanism that reduces their production, without causing uncoupling, and even leading in improved efficiency. Because of the importance of ROS as therapeutic targets, this new mechanism deserves further studies.

## Introduction

Mitochondria, because of their central role in ATP supply through oxidative phosphorylation, but also in oxygen radical production and ion homeostasis, play a crucial role in skeletal muscle function. During last decades, a growing interest has been focused on the mechanisms linking dysfunctions of the mitochondrial oxidative phosphorylation and the aging process. One of the current leading hypothesis to explain the decrease in muscle energetics with aging is the mitochondrial theory of aging. The now accepted view hypothesizes that the reactive oxygen species (ROS) production linked to the activity of respiratory chain is responsible for the damages accumulated with aging to several components of the oxidative phosphorylation machinery and mitochondrial DNA (Dirks et al., 2006; Gonzalez-Freire et al., 2015) and triggers atrophy of the muscles (Dirks et al., 2006). These damages are mainly thought to result in mitochondrial dysfunction which disables the capacity of mitochondria to fulfill cellular ATP demand during contraction (Dirks et al., 2006; Bratic and Trifunovic, 2010). Therefore ROS production and related damages are considered responsible for loss of muscle mass and function during aging.

Indeed, assessed by *in vivo* ^13^C/^31^P NMR spectroscopy at rest, a decrease of mitochondrial oxidative phosphorylation activity was found in skeletal muscle of elderly (Conley et al., 2000; Petersen et al., 2003), explained by a decrease of mitochondrial mass and activity (Conley et al., 2000). However, other authors studying the oxidative capacity in tibialis anterior did not find any modification during aging (Kent-Braun and Ng, 2000). *In vivo* in human skeletal muscle during contraction of high intensity, no dysfunction of oxidative phosphorylation was evidenced, but only a reduced participation of glycolysis (Lanza et al., 2005). Conflicting results have also been obtained on isolated mitochondria from aged muscles. The decrease in the activity of different respiratory chain complexes has been measured in skeletal muscle of aged mice (Kwong and Sohal, 2000). Maximal oxidation rate was also found to decrease during aging in skeletal muscle of mice (Mansouri et al., 2006), rats (Kumaran et al., 2005), and from biopsies of human skeletal muscle (Tonkonogi et al., 2003), as well as ATP synthesis in skeletal muscle of rats (Drew et al., 2003) and humans (Short et al., 2005). *A contrario*, other authors demonstrated the absence of alteration of oxidative phosphorylation in mitochondria isolated from human muscles biopsies (Rasmussen et al., 2003), and several other studies did not evidence any alteration in the maximal oxygen consumption rate in rats with aging (Kerner et al., 2001; Chabi et al., 2008). The absence of consensus emerging from these results obtained at different levels of integration essentially demonstrate the complexity of the mechanisms involved in aging muscle, and of their consequences on mitochondrial functions and cell energetics (Lopez-Otin et al., 2013).

These considerations emphasize again the importance of integrative approaches for the understanding of pathologies in organ functions, a step forward from isolated enzymatic activities toward regulation of biological functions. We developed for several years the top-down elasticity analysis (Brown et al., 1990; Hafner et al., 1990) of muscle and heart energetics (Diolez et al., 2007, 2015; Arsac et al., 2008), after having applied this approach on isolated mitochondria, to allow better understanding of the very mechanisms at the origin of dysfunction occurring during hypothermic to normothermic reperfusion in rat liver (Leducq et al., 1998) and the description of the effect of temperature on oxidative phosphorylation (Dufour et al., 1996).

We previously investigated mitochondrial bioenergetics *in vivo* in the gastrocnemius muscle of aged (21 months) and young adult (6 months) rats with modular control analysis and ^31^P magnetic resonance spectroscopy of energetic metabolites (Arsac et al., 2008). Because it gives real access to the integrated organ function, this approach brings out a new type of information—the “elasticities,” referring to internal responses to metabolic changes—that may be a key to the understanding of the processes involved in pathologies. We revealed by this integrative approach that the *in vivo* “elasticity” (responsiveness) of oxidative phosphorylation to the increase in ATP demand induced by contraction is significantly reduced in skeletal muscle from aged rats, a dysfunction especially marked for low contractile activity (Gouspillou et al., 2014).

We further applied the Top-Down control analysis to isolated mitochondria (Brown et al., 1990; Hafner et al., 1990) to canvass the alterations in the regulation of oxidative phosphorylation that may occur during aging. Top-down control analysis is perfectly designed to evidence modification in regulation that have effective consequences on integrated organ function, since it is insensitive to any changes that have no significant functional outcome (Brand, 1998). We use this property to determine which module(s) may be responsible for the mitochondrial dysfunctions occurring during physiological (Dufour et al., 1996) or pathophysiological (Leducq et al., 1998) events. Therefore, this approach is useful to obtain a precise description of which of the changes occurring during aging have functional consequences for the regulation of oxidative phosphorylation.

In the present paper, we show how this integrative approach evidenced an alteration in the regulation of the mitochondrial adenine nucleotide translocator (ANT) with aging. The analysis of the flow-force relationships under variable phosphorylation rates (mimicking variable cellular ATP demand) revealed that for equivalent ATP demand the energetic intermediate of oxidative phosphorylation (mitochondrial membrane potential difference, Δψ) is lower in aged mitochondria as compared to young ones. Decrease in mitochondrial membrane potential is known to reduce proton leak (Brand et al., 1994) and also ROS production (Echtay et al., 2003; Echtay and Brand, 2007; Toime and Brand, 2010). However, our results evidence that the decrease in Δψ is not due to uncoupling but interestingly to a decrease in sensitivity of ANT to ADP and appears therefore as a new mechanism for ROS production decrease induced by aging.

## Materials and methods

### Animals

Experiments were conducted on male Wistar rats aged 6 and 21 months. Rats were housed in an environmentally controlled facility (12-h/12-h light/dark cycle, 22°C) and received water and food *ad libitum* until *in vitro* experiments were performed. All experiments were conducted in agreement with the National and European Research Council Guide for the care and use of laboratory animals. P. Diolez has a permanent license to conduct experiments on animals by the “Service Vétérinaire de la Santé et de la Protection Animale du Ministère de l'agriculture et de la Forêt” (03/17/1999, license number 3308010).

### Isolation of skeletal muscle mitochondria

Male Wistar rats were anesthetized by isoflurane inhalation and killed by intraperitoneal injection of pentobarbital (60 mg kg^−1^). The GAS muscle of the right leg was then dissected and washed in the isolation medium containing 100 mM sucrose, 180 mM KCl, 50 mM Tris, 5 mM MgCl2, 10 mM EDTA, and 0.1% (w/v) BSA (pH 7.2). Before homogeinization, muscles were minced and exposed for 5 min to protease (2 mg mL^−1^ of isolation medium, Sigma P8038; Sigma-Aldrich, Saint- Quentin Fallavier, France). Mitochondria were extracted as previously described (Gouspillou et al., 2010). Mitochondrial protein concentration was determined by the Bradford method using BSA as standard (Bradford, 1976).

### Experimental determination of mitochondrial affinity for ADP

Oxidation rates were determined polarographically with a Clark electrode (Rank Brothers, Cambridge, UK). The use of a combined enzymatic system composed of glucose (5 mM), hexokinase (2.5 U mL^−1^ Sigma H4502), glucose-6-phosphatase dehydrogenase (2.5 U mL^−1^, Sigma G6378), NADP^+^ (1.6 mM) allowed the determination of phosphorylation rate since the NADPH production by this system is stoichiometrically linked to mitochondrial ATP synthesis rate. Changes in the rate of NADPH production were monitored at 340 nm by placing an optic fiber connected to a spectrophotometer in the oxygraphic vessel (Cary 50; Varian, Grenoble, France). Conversion of NADPH absorbance into NADPH concentration was performed using a molar extinction coefficient of 6.22 mM^−1^ cm^−1^. Bioluminescent assays were used to measure both ADP and ATP concentration during each experiment (Gouspillou et al., 2011). This method allowed the determination of the oxidation (KmVox) and phosphorylation (KmVp) rates affinities for ADP, as well as the determination of mitochondrial coupling efficiency as the ratio of phosphorylation over oxidation rates.

### Simultaneous monitoring of oxidation rate, membrane potential, and phosphorylation rate

Oxygen consumption, membrane potential, and ATP synthesis were monitored simultaneously (cf. **Figure 2**) in a glass vessel (final volume 3 ml) in a medium containing 140 mM sucrose, 100 mM KCl, 1 mM EGTA, 20 mM MgCl2, 10 mM KH2PO4, and 0.1% (wt./vol.) BSA (pH 7.2). Mitochondrial protein concentration used in the measurement vessel was approximately 0.3 mg ml^−1^ unless for the proton leak flux determination where the protein concentration used was approximately 0.6 mg ml^−1^. Succinate (5 mM+rotenone 2 μg ml^−1^) was used as a substrate for the monitoring of oxidative phosphorylation parameters (i.e., oxidation rate, membrane potential, and phosphorylation rate) and top-down control analysis experiments. To inhibit residual adenylate kinase activity, excess of P1,P5-Di(adenosine-5) pentaphosphate (AP5A, 20 μM) was added to the measurement medium in all experiments. State III oxidation rates were obtained by adding ADP (250 μM) and state IV oxidation rate was measured after complete ADP phosphorylation. Oxidation rates were determined polarographically with a Clark electrode (Rank Brothers) at 25°C. Concentration in air-equilibrated medium was taken as 240 μM (Dufour et al., 1996). Membrane potential was monitored using a homemade tetra-phenylphosphonium (TPP^+^) electrode coupled to an Ag/AgCl- saturated reference electrode (Tacussel Mi402; Goubern et al., 1990; Dufour et al., 1996; Gouspillou et al., 2010). The phosphorylation rate was experimentally determined by continuously monitoring the pH variations of the measurement medium (Valerio et al., 1993).

### Top-down elasticity analysis

**Figure 1:**
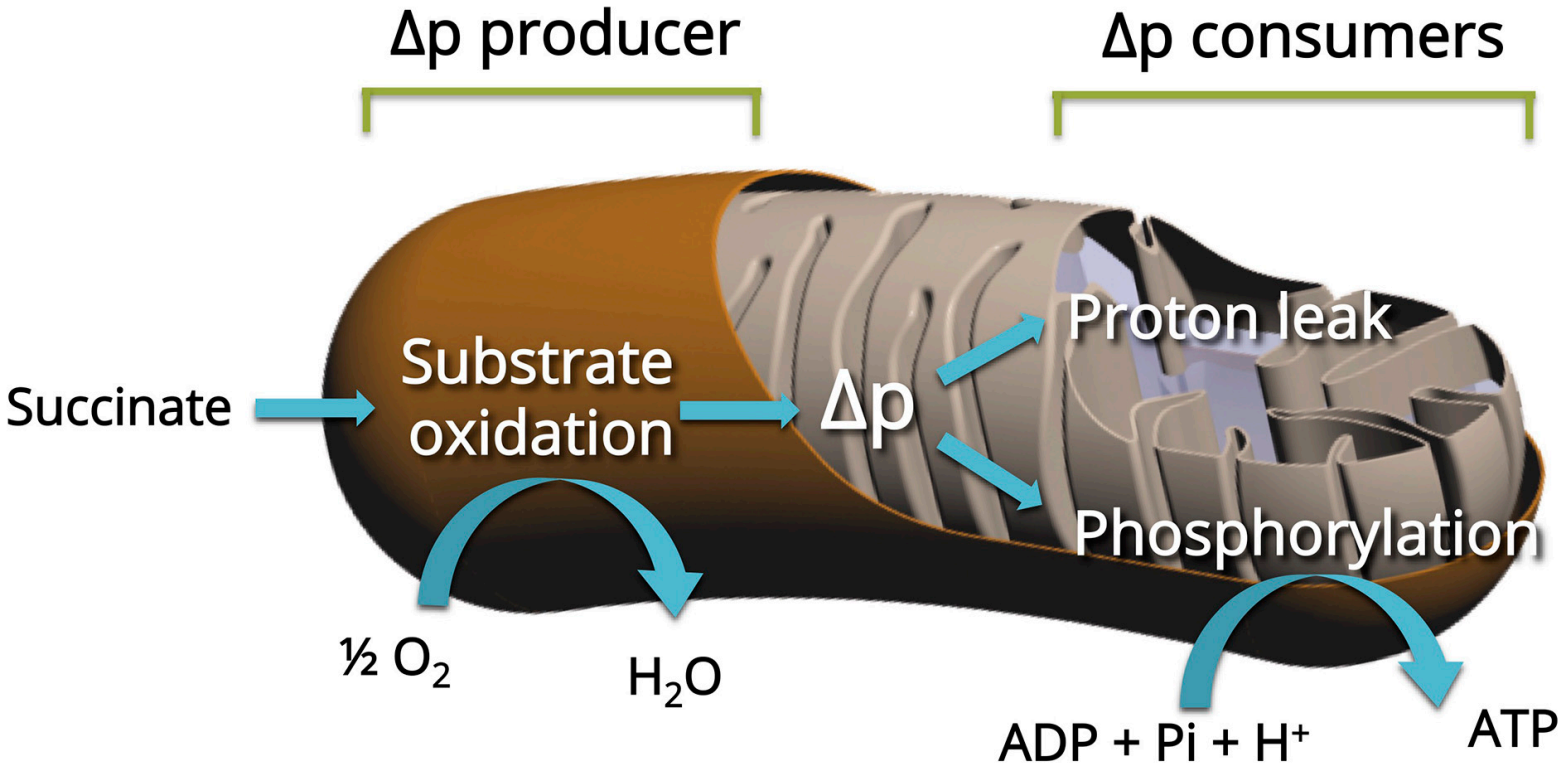
**Principle of the top-down elasticity analysis of the regulation of mitochondrial oxidative phosphorylation**. The analysis considers the electrochemical H^+^ gradient (Δp, estimated by his major component Δψ) as the intermediate between respiratory chain (Producer) and two Consumers: Proton leak and ATP phosphorylation (see Material and Methods for details).

**Figure 2:**
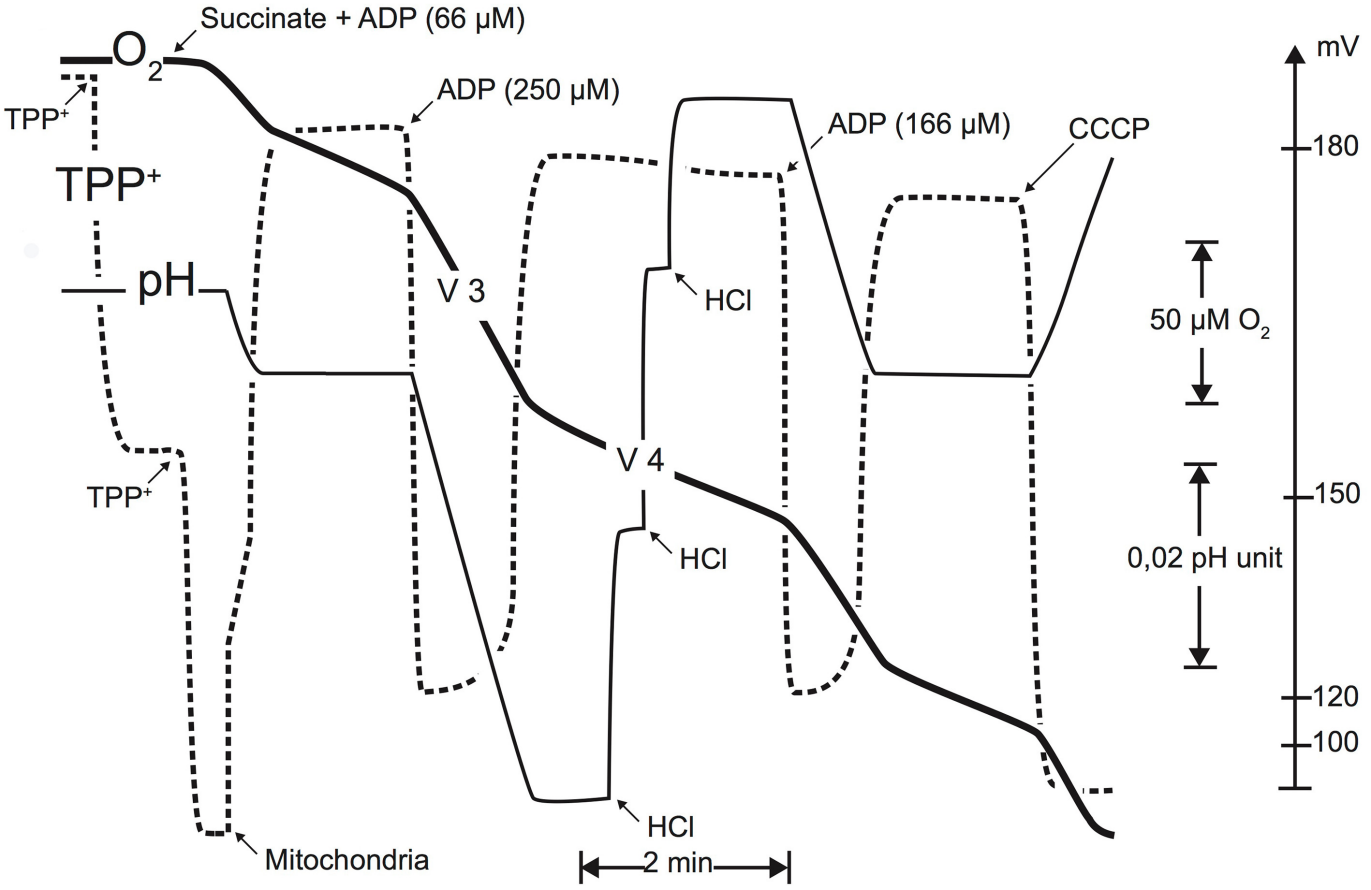
**Typical experimental simultaneous determination of oxidation rate, membrane potential (TTP^+^-specific electrode), and phosphorylation rate (pH measurement)**. These recordings were performed using succinate as a substrate (5 mM). Membrane potential was monitored using a TPP+-sensitive electrode, allowing to follow the TPP+ concentration in the medium. This electrode was calibrated by two successive additions of 1.67 μM TPP+. At the end of each experiment, an uncoupler (1 μM CCCP) was added to obtain the membrane potential baseline. Phosphorylation rate was monitored using a pH electrode, calibrated using successive additions of a titrated HCl solution (0.1 mM) (cf. Materials and Methods Section for details; figure from Gouspillou et al., 2010).

We previously applied top-down elasticity control analysis (Hafner et al., 1990) to study alterations in mitochondrial oxidative phosphorylation in mitochondria (Dufour et al., 1996; Leducq et al., 1998). Oxidative phosphorylation of mitochondria from skeletal muscle was described as three large modules linked by a common thermodynamic intermediate (Figure 1). In this system, the substrate oxidation module (substrate translocases, dehydrogenases, and respiratory chain complexes) generates the proton-motive force (Δp), the common thermodynamic intermediate, which is consumed by the phosphorylation module (Pi translocator, ANT, and ATP synthase) to produce ATP and by the proton leak module (passive permeability of the mitochondrial inner membrane to protons and any cation cycling reactions; see Gouspillou et al., 2010 for details). The top-down control analysis lies on the experimental determination of the “elasticity” coefficients over Δp (approximated by the measurement of the membrane potential Δψ)—the response to changes in Δψ—of each module. As a consequence, the complete and accurate elasticity analysis of the regulation of skeletal muscle mitochondrial oxidative phosphorylation requires the experimental determination of the flux through each of the three modules and the measurement of the intermediate concentration (Δψ) under defined conditions. Figure 2 presents a typical experiment of simultaneous determination of oxidation and phosphorylation rates with Δψ in isolated GAS muscle of an aged rat.

Elasticity coefficients of each module are calculated using modular kinetic analysis (Amo and Brand, 2007). This analysis consists in modifying the value of the intermediate (Δψ) by an adequate titration of a module that differs from the module under consideration. To cover the whole range of phosphorylation rates (from state IV to state III), these titrations were performed using succinate as a substrate at different concentrations of atractyloside in presence of an excess of ADP. The elasticity of a module toward the intermediate Δψ at a given phosphorylation rate is the relative slope of the relationship between the activity of the module and Δψ obtained with the adequate titration. The elasticity depends on oxidative phosphoryation activity and for each module we obtain an elasticity curve when expressed as a function of the intermediate (see **Figure 4**). Any change in the regulation of the module under consideration will be evidenced by a change in the elasticity curve of this very module. The complete description of the experimental protocol used to determine the kinetic response of each module to membrane potential and the calculation of elasticity coefficients for each module has been described elsewhere (Dufour et al., 1996; Leducq et al., 1998; Gouspillou et al., 2010).

### Statistics

Experimental between adult and aged rats were made using unpaired bilateral Student's *t*-tests. P values = 0.05 and 0.01 were considered significant.

## Results

**Figure 3:**
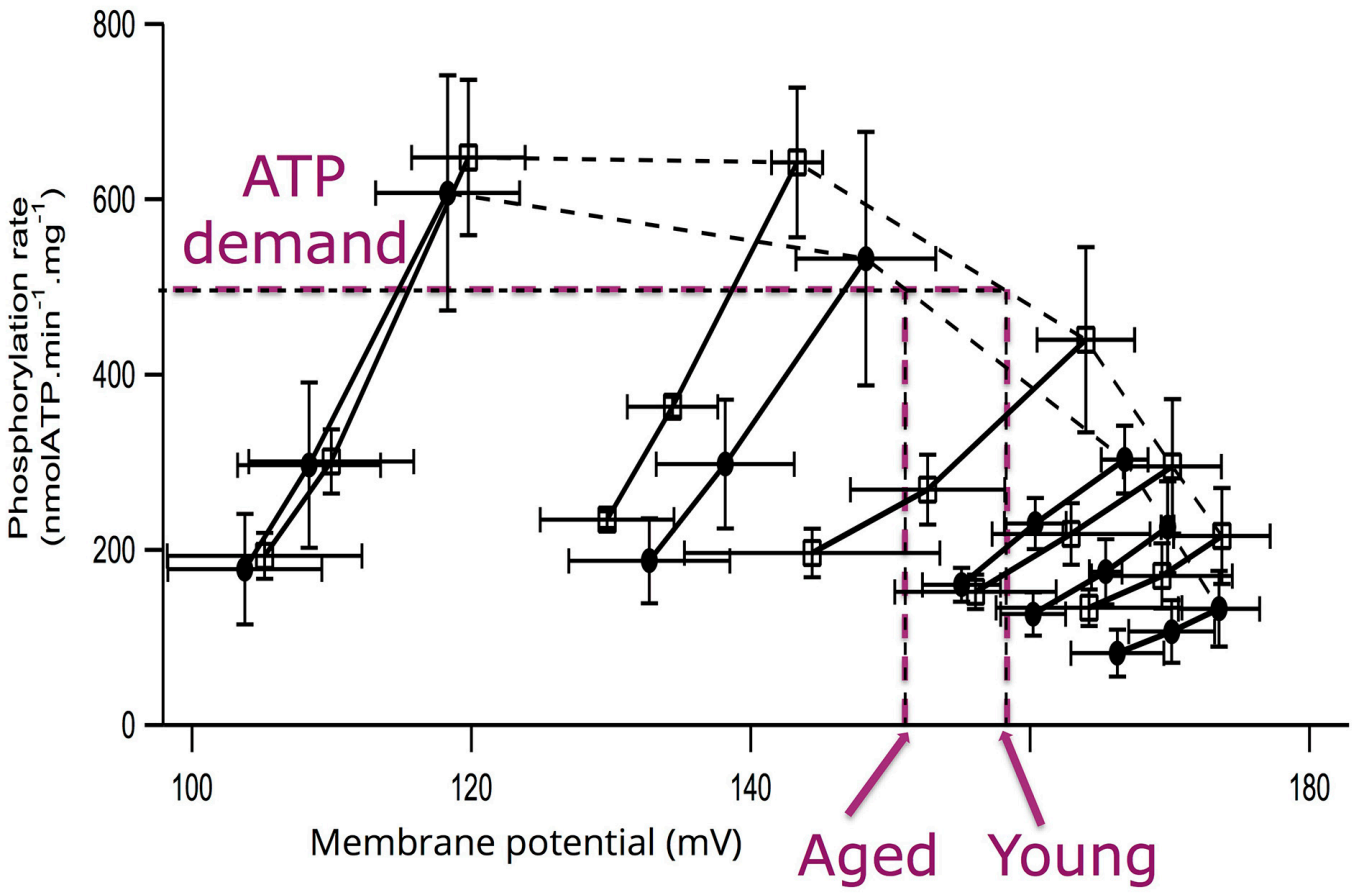
**Flow-force relationships obtained during modular kinetic analysis of mitochondrial oxidative phosphorylation**. For each measurement, saturating ADP (1.7 mM) was present in excess and succinate was used as a substrate. The different phosphorylation rates were obtained by inhibition of the phosphorylation activity obtained by adding increasing atractyloside concentration for young (squares) and aged (circles) rats. Under each condition, respiratory chain activity was further progressively inhibited by increasing malonate concentrations. Purple dashed lines exemplify the differences in membrane potential values for young and aged rats corresponding to a given phosphorylation activity (ATP demand; Data and figure from Gouspillou et al., 2010).

**Figure 4:**
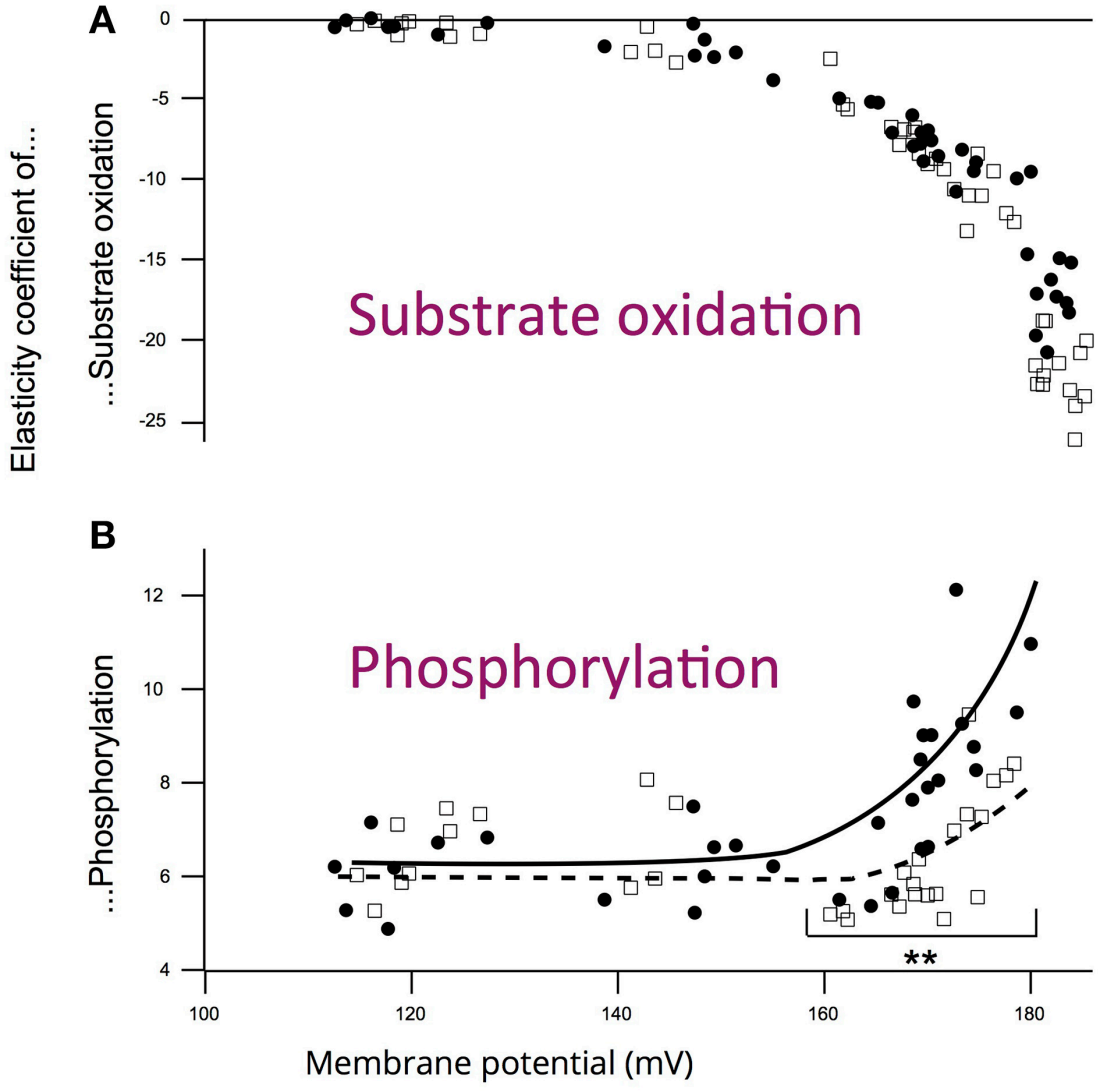
**Influence of aging on the elasticity curves of (A) substrate oxidation and (B) phosphorylation modules toward membrane potential for young (squares) and aged (circles) rats**. Data from experiments similar to those presented in Figure 3. ^**^p < 0.01 between young (*n* = 7) and aged groups (*n* = 7). Art drawing has been added to emphasize the difference between young and aged rats (Data and figure from Gouspillou et al., 2010).

The experiments performed on mitochondria isolated from aged and young rats did not evidence any difference in maximal membrane potential values (Gouspillou et al., 2010). Membrane potential values were not affected with aging, either under state III (maximal phosphorylation) or under state IV (no net phosphorylation) conditions. However, while the oxidation rate under non-phosphorylating conditions was identical between aged and young rats, the values of phosphorylating (State II) oxidation rate as well as the respiratory control ratio were significantly decreased in aged rats. Maximal phosphorylation rate was also decreased under state III conditions in aged rats (Gouspillou et al., 2010). Here, we only present the kinetic responses of the phosphorylation module to changes in membrane potential determined on mitochondria isolated from young and aged rat muscles (Figure 3). The main observation to be made here is represented by the dashed lines, which illustrate that for a given phosphorylation rate (equivalent to cellular ATP demand) the membrane potential value is always lower in mitochondria from aged rats. These data allows the determination of the elasticity coefficients of phosphorylation (Figure 4). The complete set of experiment for the determination of the elasticity of substrate oxidation has been detailed elsewhere (Gouspillou et al., 2010). We could observe that the elasticity of both modules changed with membrane potential value, but while the curves are superimposed for substrate oxidation, they appeared significatively different for phosphorylation, especially for high values of Δψ, ranging from 160 to 180 mV, corresponding to low phosphorylation activity and higher proton leak, likely to be the most common situation *in vivo*.

**Figure 5:**
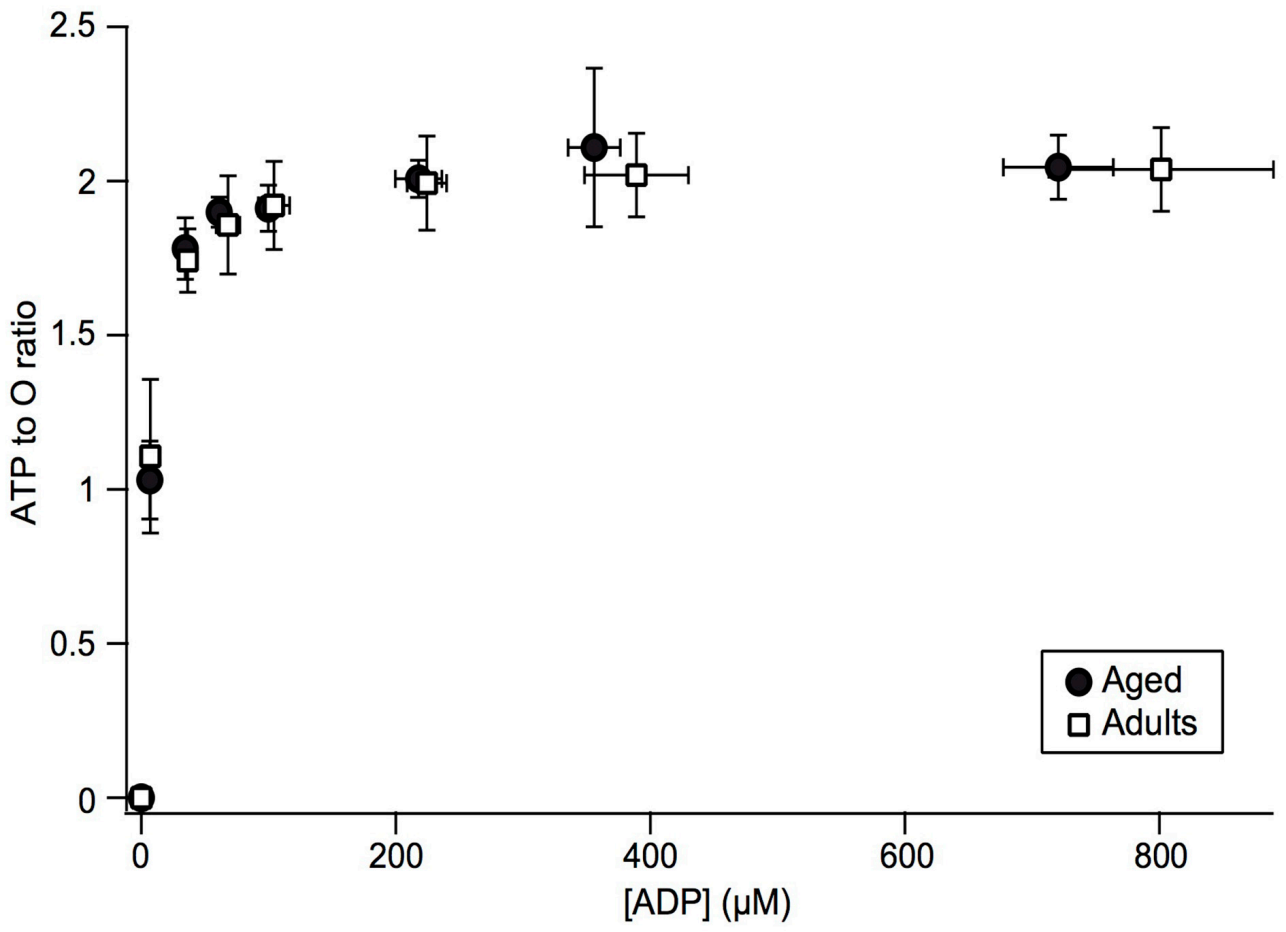
**Changes in the ATP to O ratio as a function of ADP concentration and phosphorylation rate in adult and aged mitochondria**. ATP to O ratio was determined by calculating the ratio of phosphorylation to oxidation rates and plotted as a function of ADP concentration. Data for adult (*n* = 7) and aged (*n* = 5) rats are presented as mean ± SD (Figure from Gouspillou et al., 2014).

Since a lower Δψ value is usually interpreted as uncoupling, i.e., increase in proton leak through inner mitochondrial membrane, we investigated phosphorylation coupling efficiency in mitochondria isolated from aged skeletal muscle (Gouspillou et al., 2014). This was possible thanks to our specific *in vitro* experimental setup, since we obtain simultaneous recording of oxidation and phosphorylation rates and therefore direct access to mitochondrial coupling efficiency (ATP to O ratio). ATP to O ratio was determined under conditions of constant ADP concentration across the whole range of mitochondrial oxidative phosphorylation activity (Gouspillou et al., 2014). Results are shown in Figure 5 for young and aged rat muscle mitochondria. The results demonstrate that maximal ATP to O ratio was not diminished in aged rat mitochondria. Same result was obtained concerning the dependence of coupling efficiency on the ADP concentration. Most interestingly, when the ADP to O ratio was plotted as a function of the phosphorylation rate, we found that for any given phosphorylation rate aged mitochondria displayed a (non-significative) trend for higher coupling efficiency (results not shown, Gouspillou et al., 2014).

## Discussion

The purpose of the present paper is to describe a new mechanism of protection against ROS production appearing in skeletal muscle mitochondria during aging. This mechanism involves an alteration of oxidative phosphorylation due to mitochondrial ANT remodeling and results in a decreased ROS production in aged mitochondria for a given ATP synthesis rate. The role of mitochondrial ROS production in ANT remodeling remains to be elucidated.

Being partly composed of glycolytic fibers, well known to be affected during aging (Lexell, 1995) the gastrocnemius muscle is highly sensitive to sarcopenia (Martin et al., 2007), and therefore appeared physiologically relevant for this study of mitochondrial alteration induced by aging. The current theory to explain skeletal muscle aging relies on the accumulation of oxidative damages to mitochondrial DNA and proteins, due to mitochondrial ROS production (Dirks et al., 2006; Lesnefsky and Hoppel, 2006). Since phospholipids are also important targets of ROS, it has been proposed that an increased conductance to proton may occur during aging, and therefore a decrease in Δψ. However, such decrease in Δψ would reduce mitochondrial ROS production (Brand, 2000). The increased proton leak found in aged mitochondria in some studies performed on rat (Kumaran et al., 2005) and human muscles (Tonkonogi et al., 2003) has been interpreted as a compensatory mechanism to decrease ROS production. A role for the uncoupling proteins has also been suggested (Brand and Esteves, 2005).

Thanks to integrative approaches applied at different integration levels—i.e., muscle *in vivo* and isolated muscle mitochondria—we could demonstrate that (i) the responsiveness (elasticity) of energy production is effectively reduced in the GAS of aged rats (Gouspillou et al., 2014) and (ii) that this alteration of muscle energetics is correlated with a modified regulation of phosphorylation module in mitochondria (Figure 4 and Gouspillou et al., 2014). In other words, in the aging muscle, a greater rise in ADP, concomitant with a greater drop in PCr level is required to induce equivalent activation of ATP/PCr synthesis in aged compared to young muscle. Since the elasticity coefficients are intrinsically linked to the kinetics of the modules (Brand and Curtis, 2002) and because we investigated only *in vivo* low contractile activities of the muscles—unlikely to require the maximal oxidative phosphorylation capacity, we could previously demonstrate that a decrease in maximal oxidative phosphorylation capacity (or in mitochondrial content) is unlikely to explain the decrease in the elasticity of the energy-supply module found in aged rats (Gouspillou et al., 2014). Therefore, the altered regulation of muscle energetics strongly suggest the existence of an alteration in mitochondrial function related to muscle aging and sarcopenia. This *in vivo* impairment of mitochondrial bioenergetics in aged muscle was further completed by the *in vitro* integrative study of mitochondrial bioenergetics for better understanding of the cellular basis. Indeed, while maximum oxidation and phosphorylation rates were both decreased in aged mitochondria, only phosphorylation did show modified elasticities (Figure 4).

**Figure 6:**
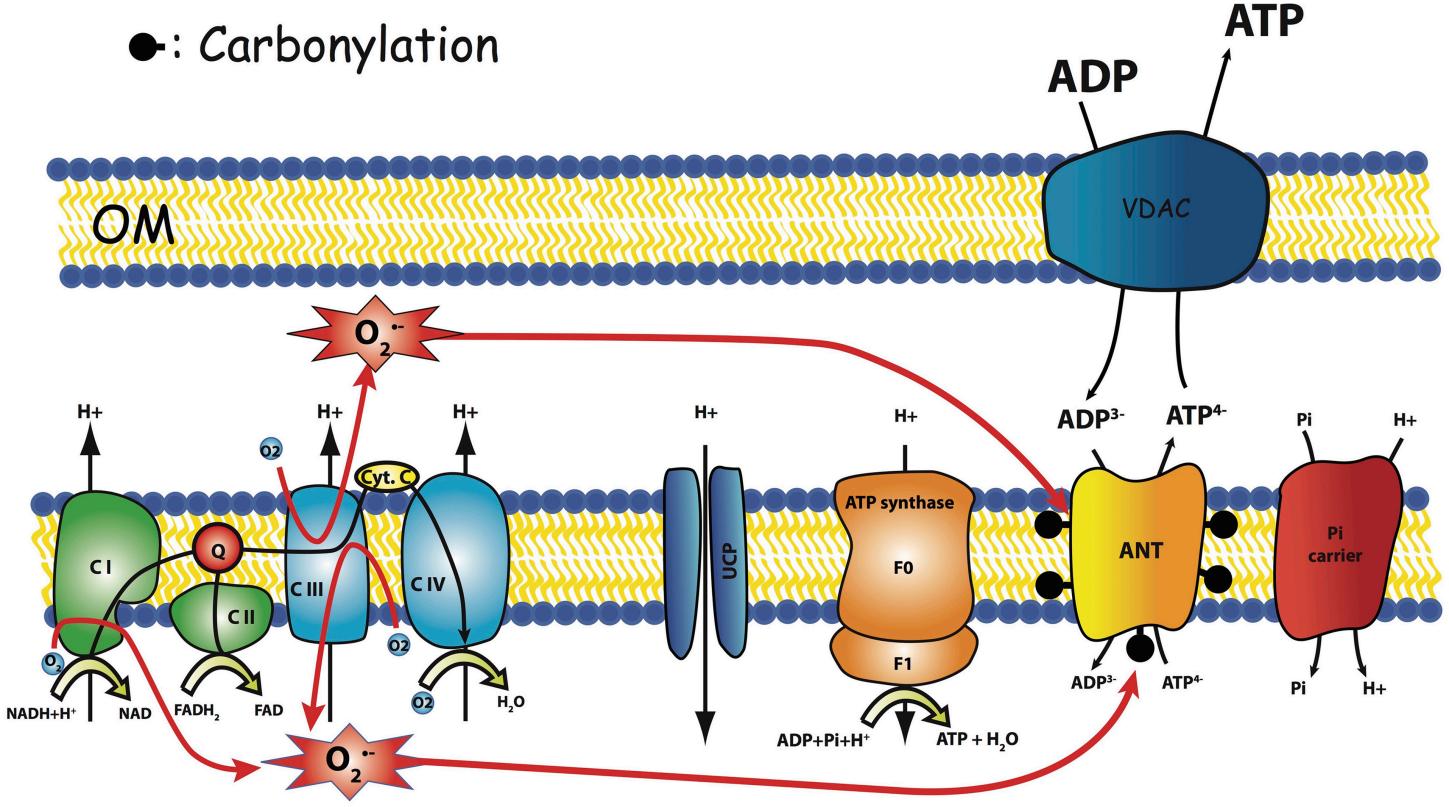
**Hypothesis**. Carbonylation of ANT as the result of respiratory chain ROS production modify ANT regulation. OM, Outer Membrane.

We recently reported a significant decrease in mitochondrial affinity for ADP in mitochondria isolated from aged GAS muscle as compared to young ones (Gouspillou et al., 2014), which is perfectly in line with the age-related reduction in the *in vivo* activation of mitochondrial oxidative phosphorylation in response to an increase in ATP demand (decrease in elasticity of energy supply). In fact, a decreased affinity for ADP indicates that for a given rate of mitochondrial oxidative phosphorylation to match ATP demand, a higher increase in the ADP concentration is necessary. This lower ADP sensitivity of mitochondria in aged rats fits well the regulation of phosphorylation (elasticity) measured *in vivo* in aged rat muscles. We also showed that mitochondria from gastrocnemius muscle of aged rat manifest an interesting increase in atractyloside-sensitivity, as compared to young ones (Gouspillou et al., 2010). Even though the apparent mitochondrial oxidative phosphorylation affinity for ADP involves several electrogenic—membrane potential sensitive—enzymatic complexes (ATPsynthase, ANT and phosphate transporter), this parameter greatly depends on ANT kinetic properties (Gouspillou et al., 2011). These results prompted us to propose that aging-related changes in mitochondrial oxidative phosphorylation implicate a modification of the functionning of ANT (Gouspillou et al., 2010, 2014). Since we also showed that ANT content was not modified in aged muscles (Gouspillou et al., 2014), we propose that a functional change in ANT characteristics may be at the origin of the aging-related change in atractyloside sensitivity and energetics remodeling in muscle. It has also been shown that ANT carbonylation, indicative of oxidative damage by ROS, was increased during aging in the flight muscle of the housefly (Yan and Sohal, 1998) and in rat skeletal muscle (Feng et al., 2008). Since the increase in the carbonyl content of ANT has been associated with impaired ANT function (Yan and Sohal, 1998), these results strongly suggest that oxidative damage to ANT with aging represents a possible mechanism that may trigger dysfunction of mitochondrial bioenergetics with aging (Figure 6).

By contrast with previous studies reporting either a decline (Tonkonogi et al., 2003; Kumaran et al., 2005; Marcinek et al., 2005) or increase (Kerner et al., 2001) in mitochondrial coupling efficiency, we did not observe any aging-related changes in proton leak, since membrane potential and state 4 oxidation rates were unchanged, as well as proton leak curve (Gouspillou et al., 2010). This absence of change in proton leak curve also clearly indicate that the age-related modification of ANT regulation described here do not affect the proton leak capacity of ANT, now considered as the primary route for proton leak in mammalian mitochondria (Parker et al., 2009; Jastroch et al., 2010). We recently added complementary data to confirm the absence of uncoupling by directly measuring the ATP/O ratio (phosphorylation efficiency) for a range of ADP concentrations driving increasing phosphorylation rates. The results confirmed in our hands the total absence of a decrease of coupling efficiency in aged mitochondria as compared to young ones (Figure 5 and Gouspillou et al., 2014). In this latter paper, we even showed that the decrease in membrane potential for a given ATP demand (phosphorylation rate) results in a tendency to increase coupling efficiency. This result is the consequence of down-regulation of proton leak by the decrease in membrane potential (Brand et al., 1994). We should however stress here that even though the decrease in Δψ by aged ANT has beneficial effect on mitochondrial ROS production, it may also have harmful consequence for muscle cells since mitochondrial membrane potential is deeply involved in ion (calcium) homeostasis.

In the framework of the radical theory of aging, these important modifications in ANT function may be the result of oxidative damage caused by intra mitochondrial ROS and may appear like a protective remodeling where ROS induce a mechanism that reduces their production, without causing uncoupling. However, further work is now required to define the origin of the impairment of ANT function with aging and to better characterize the importance on mitochondrial bioenergetics defects in sarcopenia. Because of the importance of ROS as therapeutic targets, we believe that this impairment in ANT function might be a central mechanism causing aging-related defects in mitochondrial bioenergetics which deserves further studies.

## Funding

Part of this work has been supported by the CNRS (PD) and IHU-LIRYC (Université de Bordeaux, CHU de Bordeaux) (ANR-10-IAHU-04), INSERM U1045 and the “Région Aquitaine.” GG is funded by a NSERC discovery grant (RGPIN2014-04668).

### Conflict of interest statement

The authors declare that the research was conducted in the absence of any commercial or financial relationships that could be construed as a potential conflict of interest.

## Abbreviations

GAS: gastrocnemius muscle
ANT: Adenine nucleotide translocator.
